# B-Mode Ultrasound Imaging, Doppler Imaging, and Real-Time Elastography in Cutaneous Malignant Melanoma and Lymph Node Metastases

**DOI:** 10.3390/healthcare1010084

**Published:** 2013-10-23

**Authors:** Takayoshi Uematsu, Masako Kasami, Yoshio Kiyohara

**Affiliations:** 1 The Department of Clinical Physiology, Shizuoka Cancer Center Hospital, Naga-izumi, Shizuoka 411-8777, Japan; 2 The Department of Pathology, Shizuoka Cancer Center Hospital, Naga-izumi, Shizuoka 411-8777, Japan; E-Mail: juliasandydp@yahoo.co.jp; 3 The Department of Dermatology, Shizuoka Cancer Center Hospital, Naga-izumi, Shizuoka 411-8777, Japan; E-Mail: y.kiyohara@scchr.jp

**Keywords:** cutaneous malignant melanoma, lymph node metastases, B-mode ultrasound imaging, Doppler imaging, real-time elastography

## Abstract

Examination by ultrasonography (US) is a rapid, sensitive, cost-effective, and even portable technique for confirming the presence of tumors. However, US is not routinely used worldwide for the diagnostic work-up of cutaneous malignant melanoma. High-resolution US using a 6–14 MHz or 5–13 MHz linear transducer enables the preoperative assessment of tumor size and thickness. Compared with physical examination, US is also very effective in the early detection of lymph node metastases. It can be easily repeated for the follow-up of cutaneous malignant melanoma and lymph node metastases. Ultrasonographic appearance of some lymph nodes may overlap, thus producing diagnostic pitfalls. In such cases with overlapping findings, Doppler imaging and elastography may additionally facilitate the evaluation of cutaneous malignant melanoma and lymph node metastases. US-guided fine needle aspiration cytology (FNAC) finally helps to confirm ultrasonographic results, thus improving the specificity and sensitivity in difficult situations in which US alone gives unclear results in lymph node assessment.

## 1. Introduction

In general, ultrasonography (US) has not been routinely used worldwide for the diagnostic work-up of cutaneous malignant melanoma [1]. Here, we used high-resolution US, including Doppler imaging and elastography, for the preoperative assessment of tumor size and thickness as well as for the assessment of regional lymph nodes. This article aimed to describe the spectrum of ultrasonographic findings of cutaneous malignant melanoma and lymph node metastases and correlate the ultrasonographic features of these lesions with pathological findings. By this article, we hope to demonstrate the general applicability of these techniques and encourage their wider adoption.

## 2. Experimental Section

### 2.1. Equipment

In this article, B-mode US, Doppler US, and elastography images were obtained by a radiologist (T.U.) with 20 years of experience in US using a high-resolution US machine (EUB-7500; Hitachi-Aloka Medical, Tokyo, Japan) with a 6–14 MHz or 5–13 MHz linear transducer. In addition, the radiologist also had 4 years of experience in data acquisition and interpretation of elastographic images.

### 2.2. Imaging Technique

The effective use of US for cutaneous malignant melanoma requires a thorough understanding of the technical factors influencing ultrasonographic images. Given that cutaneous and/or subcutaneous lesions are superficial lesions, penetration of the ultrasound beam is decreased because of beam defocusing. Consideration of the three major technical factors, *i.e.*, frequency, focal zone, and dynamic range, can minimize the effect of limitations, resulting in very high resolution, lesion conspicuity, and diagnostic yield.

### 2.3. Frequency

Very high-frequency US with 20–100 MHz probes has been used in dermatology [2] and has become a well-established technique for determining the vertical thickness of cutaneous tumors [3]. In addition, with tumor thickness, the likelihood of lymph node involvement increases in parallel [4]. Because cutaneous tumors, such as melanomas, require excision to a depth including the deep fat or fascia layer of the subcutaneous tissue [5], it is of crucial importance to have preoperative information of the possibility of involvement of deeper structures such as nerves and vessels. When the ultrasound frequency is increased, axial resolution improves but with a loss of image depth. This can be a problem for detecting tumor involvement of deeper structures. Moreover, lateral and longitudinal extension of a cutaneous tumor could be defined by clinical observation with a safety margin. The peripheral border or edge of a faintly pigmented or regressed melanoma may be difficult to ascertain in some patients, particularly in those with extremely sun-damaged skin. In such a case, examination of the lesion using dermoscopy or a Wood’s lamp may help delineate the periphery of these melanomas to avoid a too narrow excision of the lesion [6]. Therefore, ultrasonographic techniques in cutaneous tumors must focus more on the visualization of deeper structures in subcutaneous tissue than on the visualization of superficial structures in cutaneous tissue. High-frequency US, which uses a 5–15 MHz probe and penetrates to a depth of 40 mm, is the best method for providing crucial preoperative information regarding cutaneous tumors because it is able to investigate tumor involvement to a depth beyond the dermis. It is also useful for the assessment of lymph node involvement.

### 2.4. Focal Zone

**Figure 1:**
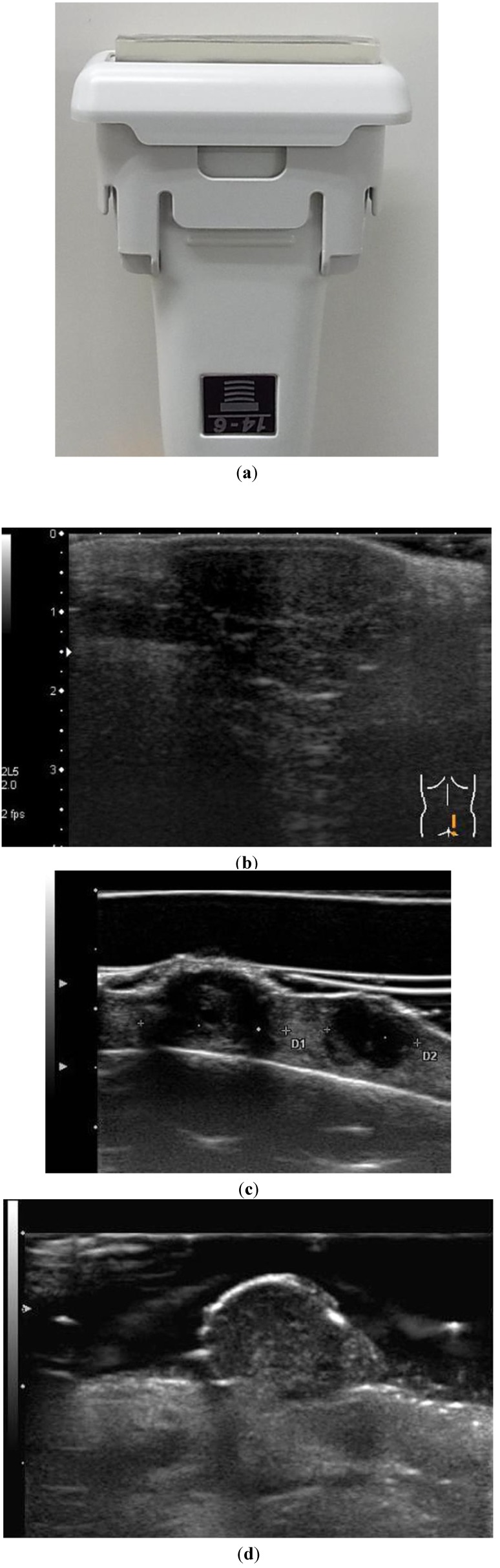
(**a**) Image shows an US probe and a gel stand-off pad. (**b**) A sonogram obtained with direct skin contact does not allow for optimal visualization of the cutaneous malignant melanoma. (**c**) **A** sonogram obtained with a gel stand-off pad allows for optimal visualization of the cutaneous malignant melanoma. (**d**) A sonogram obtained with a generous amount of US jelly similarly allows for optimal visualization of the cutaneous malignant melanoma.

Transducers must be tightly focused to optimize resolution and minimize contrast loss due to beam diffraction and defocusing. To maximize conspicuity, the lesion must be imaged within the focal zone of the transducer. Such imaging is facilitated by the use of electronic focusing for all lesions and is further enhanced by the use of a gel stand-off pad (Figure 1). Instead of using a traditional gel stand-off pad the application of a larger amount of ultrasound jelly (Figure 1) can help visualize the lesion by increasing penetration of the ultrasound beam. This is particularly useful for the superficial lesions close to the surface of the skin.

### 2.5. Dynamic Range

It is important to set the dynamic range, total gain, and time-gain compensation curve to enhance the quality of ultasonographic examination. If the dynamic range is too narrow, hypoechoic lesions will appear anechoic, and if it is too wide, lesions may appear isoechoic and may not be detectable on US. Total gain can accentuate the effects of an inappropriate dynamic range. The time-gain compensation curve should be adjusted to gradually increase with increasing depth and equalize the echogenicity of fatty tissues at all subcutaneous depths.

## 3. Results and Discussion

### 3.1. Ultrasonographic Appearance of Normal Skin

**Figure 2:**
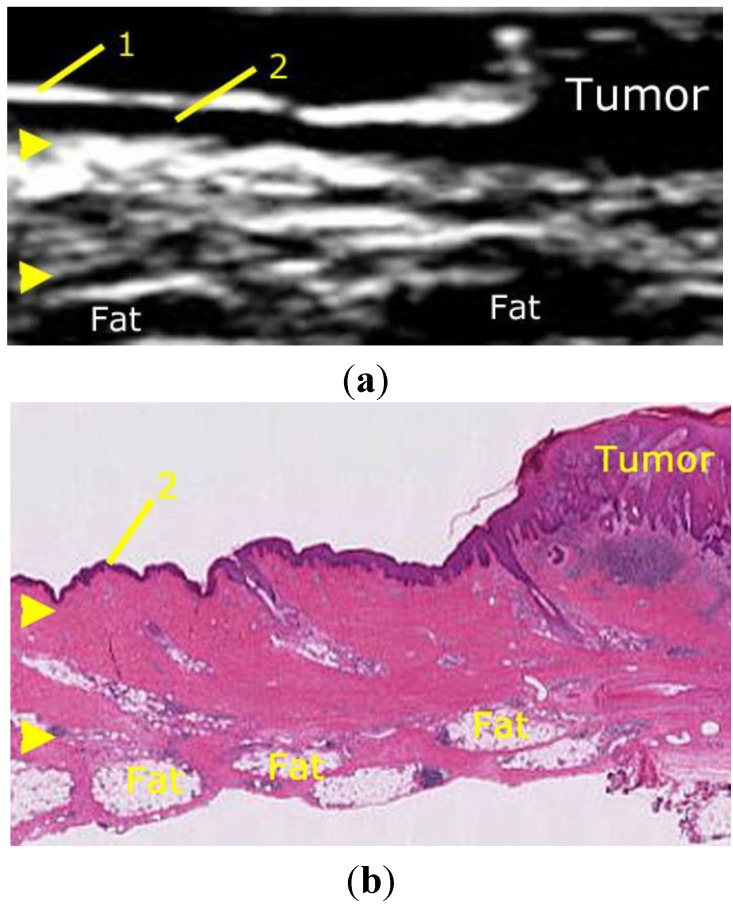
(**a**) Sonographic appearance of normal skin using a 6–14 MHz linear transducer. The skin shows the three following layers: 1, interface between the epidermis and the ultrasound jelly or gel stand-off pad; 2, the dermis; arrowheads, interface between the dermis and the subcutaneous tissue. The tumor was *in situ*; therefore, the first hypoechoic layer became thick. (**b**) The histologic section almost corresponds to the sonographic image shown in (**a**).

The skin is composed of the three following layers: the epidermis, dermis, and subcutaneous tissue. Three distinct layers can be depicted on US using a 6–14 MHz linear transducer as follows (Figure 2): a first hyperechoic layer, which corresponds to the interface between the epidermis and the ultrasound jelly or a gel stand-off pad; a first hypoechoic layer, which is influenced by high cell density and corresponds to the dermis; a second thick hyperechoic layer, which corresponds to the interface between the dermis and the subcutaneous tissue because of its content of connective tissues such as collagen and matrix.US cannot depict the epidermis itself.

### 3.2. Assessment of Cutaneous Malignant Melanoma

**Figure 3:**
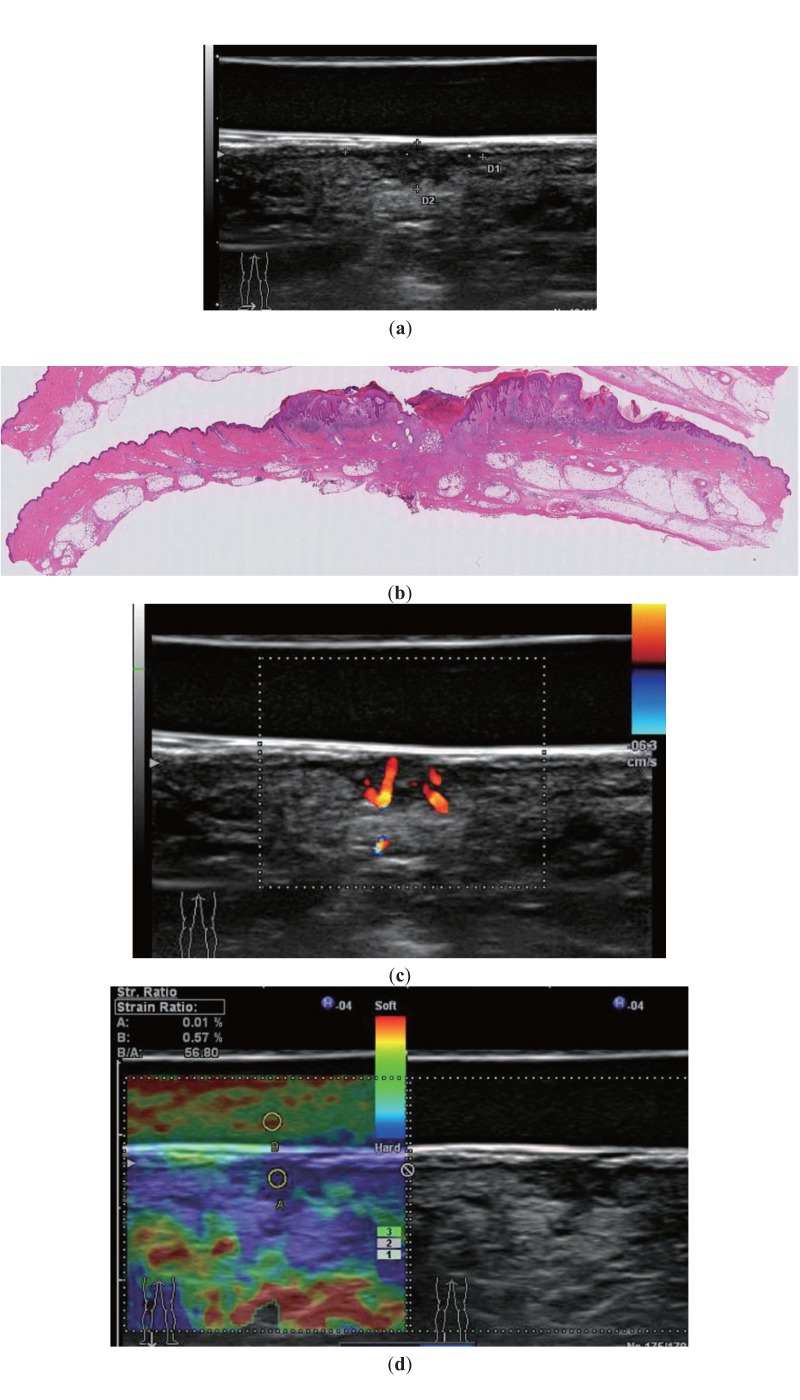
A 72-year-old man with cutaneous malignant melanoma of the sole. (**a**) B-mode US with a gel stand-off pad shows an 8.1 (wide) × 3.9-mm (deep) irregular fusiform hypoechoic lesion (between markers) that involves the epidermis, dermis, and subcutaneous tissue. The vertical thickness measured by US directly corresponds with that measured by histological examination. (**b**) Photomicrograph (Hematoxylin and eosin staining (H&E, ×1)) of a histologic specimen shows malignant melanoma with ulceration involving subcutaneous tissue. Histopathological tumor thickness is 4.0-mm. (**c**) Color Doppler US with a stand-off pad shows increased blood flow within the same lesion. (**d**) Elastography shows almost the entire lesion as blue with the strain index of the mass as 56.80, calculated as the stand-off pad strain 0.57 divided by the lesion strain 0.01.

The sonographic measurement of the vertical thickness has a relevant practical value (Figure 3) because presurgical measurement enables a one-time excision of the lesion without the need for repeated wide resection in the case of thick malignant tumors. However, the vertical thickness measured sonographically sometimes does not directly match with that measured histologically because of tension loss and dehydration of the excised material and the unique possibility that histology can discriminate between tumor and reactive tissue, which is not possible by performing mere US [7].

### 3.3. Doppler Imaging

Tumor growth depends on neovascularization. The Doppler signal is useful in the diagnosis of cutaneous malignant melanoma because it recognizes intralesional color and correlates it with the Breslow index and patient survival (Figure 3) [3,4]. Angiogenesis is also an important factor in the metastasis from cutaneous malignant melanoma. Therefore, it is important to examine tumor vascularity and depict it using Doppler imaging combined with B-mode US. In particular, peripheral perfusion is an early sign of involvement and of crucial importance for achieving a high identification rate of sentinel lymph nodes metastases [8].

### 3.4. Elastography

Real-time elastography, a technology in its infancy, is based on the principle that softer, normal tissue deforms more easily than malignant tissue, which is generally stiffer [9]. Elastography is a dynamic technique that estimates tissue stiffness by measuring the degree of distortion under the application of an external force. A recent study reported that elastography can enhance the diagnostic accuracy of US for differentiating between reactive and malignant lymph nodes in cutaneous malignant melanoma and may eliminate the need for sentinel lymph node biopsy [10]. At present, two principal elasticity imaging methods are available for evaluating lesions [11]. The elastic strain ratio is a semiquantitative method that reveals the stiffness of tissue and identifies benign and malignant lesions by comparing the difference in compliance between the lesion area and the normal tissue surrounding the area. Hence, the elastic strain ratio is a more objective method to differentiate lesions [11]. In our study, to evaluate the elastic strain ratio, we compared the difference in compliance between a cutaneous/subcutaneous lesion area using a gel stand-off pad or a generous amount of ultrasound jelly. When measuring the elastic strain ratio, the region of interest (ROI) of a lesion is generally located in the contour of the lesion, whereas the corresponding ROI in the gel stand-off pad or the generous amount of ultrasound jelly is selected as a control to avoid inter-patient tissue variations (Figure 3). Our method may be a more objective analysis for evaluating the elastic strain ratio in cutaneous/subcutaneous lesions. Therefore, we believe that with further refinement, elastography has the potential to become a useful noninvasive tool for the diagnosis of cutaneous tumors.

### 3.5. Assessment of Lymph Nodes

**Figure 4:**
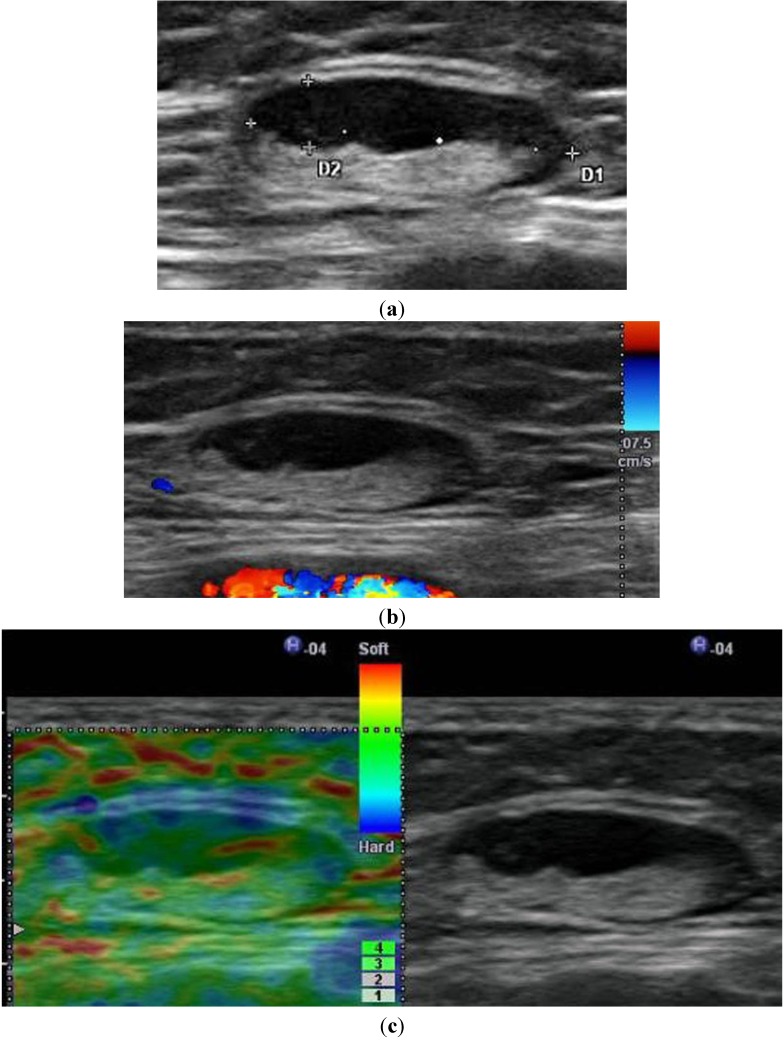
A 53-year-old woman with cutaneous malignant melanoma of the sole. No lymph node metastasis was confirmed histologically. (**a**) B-mode US shows a 16.9 × 6.7-mm enlarged lymph node of the inguinal region with an eccentric broadening of the parenchyma and a withdrawal of central echoes to one side (bottom). (**b**) Color Doppler US shows no central and peripheral perfusion. (**c**) Elastography shows a mosaic pattern of green and red. The lymph node can be estimated as being soft on the basis of this mosaic pattern. Elastography can provide a benign finding.

**Figure 5:**
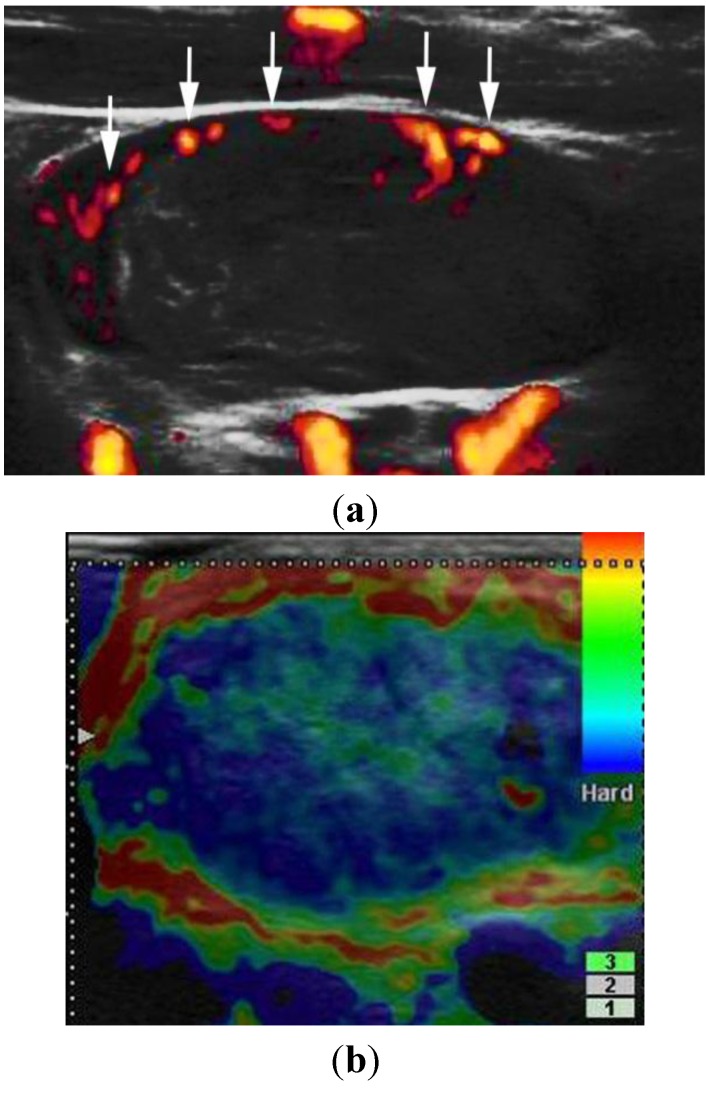
A 65-year-old woman with cutaneous malignant melanoma of the nose. Massive metastasis was confirmed histologically. (**a**) Power Doppler US shows increased vascularity with peripheral perfusion (arrows). Note an 11.4 × 5.1-mm enlarged lymph node with the absence of echogenic hilum. (**b**) Elastography shows almost the entire lesion as blue. Elastography can provide a malignant finding.

**Figure 6:**
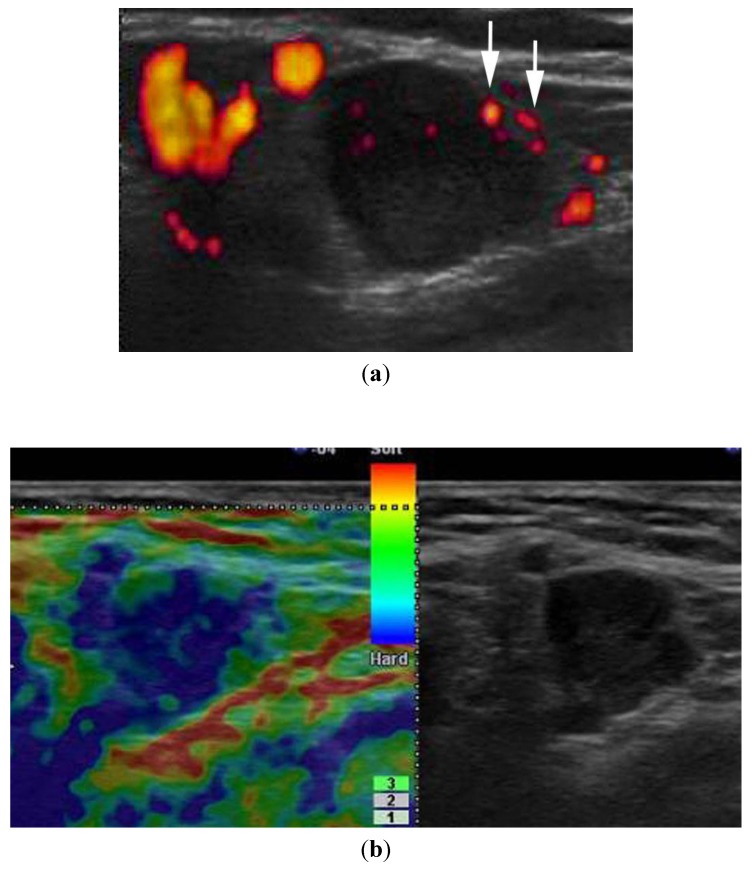
A 76-year-old woman with cutaneous malignant melanoma of the nose. Cervical lymph node metastasis was confirmed histologically. (**a**) Power Doppler US shows increased vascularity with peripheral perfusion (arrows). Note a 9.9 × 11.5 mm balloon-shaped enlarged lymph node. (**b**) Elastography shows almost the entire lesion as blue. Elastography can provide a malignant finding.

Examination of regional lymph nodes (Figure 4, Figure 5 and Figure 6) by US plays an important role in the preoperative treatment and follow-up of patients with invasive cutaneous melanoma [8,12]. Ultrasonographic descriptors for suspicious lymph nodes are increased, namely, vascular signature, rounding of the node (*i.e.*, loss of its normal ovoid shape), loss of the normal hilar echoes and their replacement with low-level internal echoes, and the presence of focal low-level subcapsular space echoes or asymmetric widening of the subcapsular space [8]. Voit *et al.* reported that the most important criterion of lymph node involvement is the loss of central echoes (Figure 5) and/or a balloon-shaped appearance (Figure 6) of the lymph node [8]. Peripheral perfusion (Figure 5 and Figure 6) is an early sign of lymph node involvement [8]. If it is suggested by ultrasonographic findings that sentinel lymph node contains metastatic disease, it is useful to confirm this by performing US-guided FNAC [12]. US-guided FNAC of sentinel lymph nodes is a highly accurate method to identify positive sentinel lymph nodes without sentinel lymph node biopsy [12]. Therefore, it has the obvious benefit of reducing the number of sentinel lymph nodes surgically biopsied, thereby reducing the cost and the associated morbidity. Ultrasonographic examination is highly operator-dependent, thus making it difficult to reproduce the assessment of lymph node involvement. However, US-guided FNAC of sentinel lymph nodes can finally overcome the limitations of US because it can definitely confirm a metastasis that was suspected by US.

### 3.6. Locoregional Spread

Malignant melanoma metastases are described as satellite metastases when they are found 2–3 cm from the primary tumor and as in-transit metastases when the lesion is found at a greater distance (>3 cm) along the lymphatic course toward the locoregional lymphatic basin [13]. US is more sensitive and specific than palpation for detecting satellite and in-transit lesions [13].

## 4. Conclusions

High-resolution US is an invaluable tool for imaging in dermatology. It can provide crucial preoperative information by serving as a first-line examination for both thickness and characterization of cutaneous malignant melanoma. Compared with a physical examination, US is very effective in the early detection of lymph node metastases. US combined with elastography and color/power Doppler imaging may also be more helpful in the evaluation of cutaneous malignant melanoma and lymph node metastases. US-guided FNAC helps to improve specificity and sensitivity in difficult situations in which US alone gives unclear results.
